# Occupational Lives in Sustained Recovery From Alcohol Dependency: An Interpretive Phenomenological Analysis

**DOI:** 10.1177/15394492211042265

**Published:** 2021-09-07

**Authors:** Emily L. Vegeris, Rob Brooks

**Affiliations:** 1 Leeds Beckett University, UK

**Keywords:** occupation, qualitative research, health, leisure

## Abstract

Alcohol use is a significant risk factor for ill health. Although many people complete treatment, only one third maintain their sobriety. Research has suggested that occupational engagement supports early recovery, but its role in sustained recovery is not known. This study aimed to explore the occupational lives of individuals in sustained recovery from alcohol dependency. An interpretive phenomenological analysis methodology was used, utilizing an occupational perspective. Participants were recruited from a substance use recovery center. Data were collected through interviews and analyzed thematically. This study had three participants. Four themes emerged: (a) shaping occupational lives, (b) shifting coping strategies, (c) finding a gateway to new social connections and a sense of belonging, and (d) establishing a new role in the context of recovery. Findings suggest that in sustained recovery, occupations develop new meanings and purposes that have the potential to support recovery.

## Introduction

Alcohol use has been identified as the greatest risk factor for death, disability, and ill health for 15- to 49-year-olds (National Drug Treatment Monitoring System, 2018). In England, it has been estimated that alcohol use costs health services £3.5 billion per annum (Public Health England, 2016). In addition, the COVID-19 pandemic has led to elevated consumption of alcohol (Alcohol Change UK, 2020), leading to concern about its long-term socioeconomic impact on society. About 60% of those who enter treatment for alcohol dependency successfully completed it (Public Health England, 2019), but it is estimated that more than three quarters relapse within a year (Thompson, 2016). Although a standardized definition of recovery remains absent from the literature, there are a common ideas such as being free from dependency (National Treatment Agency for Substance Misuse, 2012), total abstinence (Laudet, 2007), and having health, a place to live, a purpose, and a community (Substance Abuse and Mental Health Services Administration, 2020). Public Health England (2019) notes each re-presentation to treatment services to be a separate “recovery journey.” In the present study, recovery will therefore be operationalized as the time that has elapsed since the individual’s most recent successful completion of substance use treatment, thus allowing exploration of their most recent recovery journey. The Betty Ford Institute Consensus Panel (2007) categorizes recovery into three stages: early recovery (the first 12 months), sustained recovery (between 1 and 5 years), and stable recovery (longer than 5 years).

Current research from an occupational perspective has considered the experience of the occupations of addiction (Wasmuth et al., 2016), the effect of an occupation-based intervention (Wasmuth & Pritchard, 2016), and occupational performance as an outcome to a peer-based program (Narain et al., 2018); these studies have included participants with substance use experience, including alcohol and drugs. No research has been devoted specifically to alcohol dependency or to sustained recovery, which this study addresses.

### Losses Attached to Cessation

Wasmuth and colleagues (2014) proposed that addictions are occupations within their own right as they shape roles, routines, behavior, and contribute to one’s occupational identity. Therefore, upon cessation, a major occupational role is lost, akin to an occupational transition (Nhunzvi et al., 2019). Recovery may evoke feelings of loss for the addiction, including associated routines and social connections (Martin et al., 2011). Loss or change of identity, role confusion, and reduced motivation may also arise (Wasmuth et al., 2014). Developing new patterns of occupation, in the form of roles, routines, and connections, which are congruent with the construction of one’s newfound occupational identity, is particularly important in mitigating these losses and supporting recovery. Luck and Beagan (2015) suggest that the occupational lives of individuals in early recovery are turbulent, encompassing lost and newfound roles and structures and Narain et al. (2018) identified the importance of a new identity and occupational roles.

### Changes in Occupational Engagement

Andersson et al. (2012) explored the relationship between women’s patterns of everyday occupation and alcohol consumption. Results indicated occupational deficits, such as an absence of self-care and rest occupations, to be positively correlated with problematic alcohol consumption. Similarly, Wasmuth et al. (2016) identified diminished occupational roles and routines in veterans with a diagnosis of substance dependence.

A desire to reengage with work, hobbies, and self-care was reported by working-age adults entering treatment for substance use (Duncan et al., 2019), and reengagement in leisure occupations has been linked with greater success in early recovery in veteran populations (Wasmuth & Pritchard, 2016). This reengagement facilitates recovery through enabling individuals to experience a new state of occupational balance, which was lost during the throes of addiction (Duncan et al., 2019).

In line with the concept of addiction-as-occupation, patterns of occupational engagement can influence and contribute to one’s occupational identity (Narain et al., 2018; Wasmuth et al., 2014). Occupational adaptation, where an individual reconstructs a positive occupational identity and achieves competency within their environment (Taylor & Kielhofner, 2017), is therefore implicated within this context. Mothers have been shown to benefit from making changes to their occupational engagement with regard to treatment completion for substance and alcohol dependency (Martin et al., 2011) and woman in substance use recovery found a transformation in their identity (Narain et al., 2018). These findings implicated the importance of developing a new and robust occupational identity which stems from changes in occupational performance patterns and repertoires. Access to new occupations, roles, and routines are therefore considered key in supporting early recovery through the reconstruction of identity (Martin et al., 2011).

### Sustained Recovery as an Occupational Transition

Studies have identified genetic components influencing relapse rates (Vengeliene et al., 2014); however, occupation-based factors can also be considered. Sustained recovery from addiction has been conceptualized as an occupational transition. Luck and Beagan (2015) explored the transition from smoking to nonsmoking. Engagement in meaningful occupations was found to support the development of a new identity as a nonsmoker, which in turn prompted the participants to engage in occupations congruent with their new identities. This change was subject to a gradual accruement of alternative ways to manage stress and engage in social and leisure activities without substances. In addition, Nhunzvi and colleagues (2019) explored recovery from alcohol dependency as an occupational transition through the lived experiences of three young Zimbabwean men. Findings highlighted ongoing participation in meaningful occupations, which linked to changes in occupational identity and engagement, as being central to recovery maintenance. Critically, ongoing participation in meaningful occupations was implicated as being imperative to maintaining newly constructed occupational identities and recovery.

### Social Environment: Risk and Protective Factors

Changes in social environment are significant in the context of recovery. While addictions can foster social connections with others of the same habit (Nhunzvi et al., 2019), the *Diagnostic and Statistical Manual of Mental Disorders* (5th ed.; *DSM-5*; American Psychiatric Association, 2013) lists interpersonal problems being exacerbated or caused by alcohol usage as a diagnostic criterion for alcohol use disorder. Friendships with others who share the goal of recovery has been found to support early recovery from substance dependencies within treatment centers (Duncan et al., 2019). In addition, a desire to rekindle lost connections with family serve as a motivator to maintain in the early stages of recovery (Bell et al., 2015).

In summary, existing research of addiction recovery from an occupational perspective has identified the potential for occupational engagement to lead to new roles and identity but has focused on early recovery in treatment settings. There is a lack of research exploring the occupational lives of individuals in sustained recovery. In addition, studies have grouped alcohol and substance dependencies as one phenomenon, despite differing rates of relapse (National Drug Treatment Monitoring System, 2018). Therefore, the aim of this study was to explore the occupational lives of individuals in sustained recovery from alcohol dependency only, and the role of occupation in sustaining recovery.

## Method

### Design

Interpretive phenomenological analysis (IPA) was employed using a double hermeneutic approach (Smith et al., 2009). IPA posits that lived experiences can be understood through examination of the meaning that individuals attach to their experiences and requires the researcher to interpret this meaning through their own theoretical lens (Smith et al., 2009). The researcher used an occupational perspective, which Njelesani and colleagues (2014) described as a “. . . a way of looking at or thinking about human doing” (p. 226). IPA enabled the researcher to describe, explore, and interpret participant perceptions of factors influencing their recovery, and to explore the nature, meaning, and purpose of occupations that participants had engaged in throughout their experiences, from an occupational perspective.

### Recruitment

Participants were recruited through purposive sampling (Clarke, 2009) by a gatekeeper; this is a common method to obtain a homogeneous IPA sample (Smith et al., 2009). Recruitment was capped at four participants to ensure detailed accounts of individual experiences, in line with IPA-recommended sample size (Smith et al., 2009). Four individuals expressed an interest, one of which withdrew prior to interview. Participant demographics are outlined in Table 1. All participants shared the lived experience of sustained recovery from alcohol dependency and pseudonyms were given to maintain anonymity. Participants were recruited from a not-for-profit substance use recovery center based in northern England. The organization does not include an occupational therapy service but offers drop-in support and activities. Participant inclusion criteria were 18 years of age or older, living in fixed accommodation, received treatment for alcohol use only, and considered self to have been in recovery from alcohol dependency for a minimum of 12 months.

**Table 1.** Participant Demographics.

Pseudonym	Age (years)	Race	Time spent in recovery (months)
Sally	49	White British	21
Michael	47	White British	13
Nigel	52	White British	16

### Ethical Considerations

Ethical approval was granted by Leeds Beckett University. The gatekeeper verbally introduced the study to potential participants and provided the researcher’s contact details. Interested participants contacted the researcher directly by email. Upon contact with the researcher, interested individuals were provided with participant information and consent forms and were given time to consider their voluntary participation. Informed consent was obtained verbally prior to interview. All participants had the right to withdraw up to the beginning of data analysis. All data were anonymized and securely stored.

### Data Collection

Semi-structured in-depth interviews were used to collect detailed, rich data through a first-person account of thoughts, feelings, and stories (Smith et al., 2009). Interviews were conducted in English via telephone and audio recorded, lasting between 45 and 60 min. Elements of the Model of Human Occupation (Taylor & Kielhofner, 2017) were used to develop the interview schedule. The interview questions/topics are presented in Table 2. The questions encouraged exploration of the individuals’ past and current occupational participation and engagement within general societal contexts and addressed participant perceptions of factors that had influenced their sustained recovery.

**Table 2.** Interview Questions.

• Can you tell me a little bit about yourself, such as how long you have been coming to the recovery center and what it is that you enjoy about being here? • I understand that you are now in recovery and that before coming here you will have been on a treatment program. Can you tell me a little bit about that? • What factors do you think have played the biggest part in maintaining your recovery? • Have there been any particular obstacles that you have needed to overcome since the end of your treatment? • How were you spending your time in the 6 months before you received treatment for alcohol use? • What was the balance between spending time with other people and being on your own before treatment? • What was it that helped you to decide to go into treatment? • Do you now have a typical daily/weekly routine in the way that you spend your time? • Do you currently have any roles or responsibilities? For example, work/voluntary/familial/student? • Have you developed any new interests/rediscovered any old interests while you have been in recovery? • Has your social circle changed in any way since you went into recovery? • What is most important to you now? • What do you think has been the driving factor for you to maintain your recovery? • What advice would you give to somebody starting their journey to recovery?

### Data Analysis

Interviews were transcribed verbatim. In line with the double hermeneutic approach of IPA, an occupational perspective was taken to analyze the data. The analysis consisted of an iterative and inductive cycle (Smith et al., 2009). The researcher made descriptive, exploratory comments to gain an understanding of participant perspectives of their experiences. Conceptual annotations were then made, which were stimulated by, and tied to, the text. A mind-mapping process was utilized to cluster potential themes in each interview; this allowed for superordinate themes to be identified for each interview. Superordinate themes of the three interviews were collated to identify connections, and idiosyncrasies, between cases. This allowed progression from the particular to the shared (Smith et al., 2009).

### Rigor, Trustworthiness, and Credibility

IPA cautions against member checking (Smith et al., 2009). Instead, the researcher engaged in discussions with individuals with lived experience of the phenomenon prior to construction of the interview schedule to promote credibility and trustworthiness. An audit trail was kept throughout the research process to enhance credibility (Cope, 2014). To promote rigor, reduce bias, and enhance consistency, the researcher (EV) regularly discussed the coding and themes with the researcher (RB). In addition, a reflective diary was used throughout the research process; these notes were referred to throughout analysis to facilitate the researcher’s self-awareness of any preconceptions or influences that may influence data collection or analysis (Darawsheh, 2014).

## Findings

Four main themes emerged from the data and are presented with participant quotes.

### Shaping Occupational Lives

This theme represents the way in which addiction appeared to influence the occupational lives of participants. All subjects experienced occupational imbalance throughout their addiction although this manifested itself in different ways. For Michael and Sally, imbalance presented as diminished interests and leisure pursuits: “I used to go watch me son, me son played rugby, too . . . I stopped going to watch him. Everything, all my interests had sort of stopped really” (Michael):

All I did was get up in a morning and first thing I do was go to t’ shop and drink twenty-odd; I could drink all day to the next day, go to sleep for a few hours, wake up and drink again. (Sally)

In contrast, Nigel appeared to neglect his self-care, identifying a lack of downtime as a risk factor in previous relapses: “I keep busy but that’s been my downfall in the past, certainly work-wise; I’d never stop, burn the candle at both ends.” Indeed, Nigel explained that he now prioritizes self-care to support his well-being.

Addiction influenced participants’ social environments. They each reported loss of significant relationships and drinking alone with little social interaction: “I would have a drink and just stay on me own all time, just locked me door and just stayed in” (Sally); “In the months before rehab [my daughter] hardly ever came round . . . only people I seen were in’t supermarket when I were going to get drink” (Michael). Furthering this, Michael reflected on the shallow nature of the relationships he developed during his addiction, which provided a contrast with his prior friendships: “They’re not real friendships, are they? . . . They’re people that you use, and they use you at the time, you know . . . all me actual real friends had gone by then.” Conversely, Nigel maintained contact with a small number of friends while hiding his addiction, explaining that he would “never tell them” through fear of judgment. When Nigel disclosed his addiction to his friends, he perceived that these relationships became stronger, further emphasizing how his addiction previously led to disconnect: “just letting everybody know what I’m going through, if anything it’s brought us all closer.”

### Shifting Coping Strategies

Addiction appeared to hold meaning and purpose for all participants; they used alcohol as a coping strategy for psychological distress but the mechanisms for coping changed in recovery. All participants explained that they drank to experience transient relief from negative affective states: “Everything got on top of me, I just started drinking and drinking and just tried to forget my past” (Sally); “I were upset but I just drunk more then ’cos I were feeling sorry for myself” (Michael); and “Drink for me is like a partial suicide; it just knocked me out completely for a while and made me forget” (Nigel). In recovery, however, all participants spoke of reengaging with new and rediscovered occupations. Crucially, these occupations held the same purpose that was once attached to alcohol: They served as coping mechanisms. Exemplifying this, Nigel described rekindling his love of song-writing that provided a medium to articulate feelings surrounding addiction: “I find it therapeutic and it’s something that I do with my friend that I met at [the organization]—we write songs together about things we’re going through.” This provides a stark contrast to Nigel’s previous secretive nature surrounding his addiction and emphasizes a newfound ability to discuss his inner struggles. Similarly, Michael explained that he attended the organization as a strategy to “[look] after me own head.” Michael also spoke of spending time with his grandson, Sam, whom he had little contact with prior to recovery. Engaging in co-occupations that nurture this relationship, such as rugby, can be seen as a key coping mechanism—one which supports Michael’s recovery by reminding him what he stands to lose: “he’s like my best mate now *laughs* so yeah, I just think to myself if I start drinking again now, I’ll lose all that again.” Similarly, Sally explained that when knitting or reading, she’s “in a world of me own,” that her “mind’s blank,” and that she can “switch off from everything that goes on,” suggesting that Sally experiences occupational flow. Crucially, these occupations can be seen as explicitly replacing the purpose of alcohol, which she previously engaged in to distract from troublesome thoughts.

### Finding a Gateway to New Social Connections and a Sense of Belonging

All participants identified the social support received at the recovery center as a key supporting factor in their sustained recovery. The organization provided access to people in similar life circumstances of recovery, within an environment devoid of stigmatization surrounding addiction. This cultivated feeling of acceptance, the development of new friendships, and reciprocated support, leading to diminished shame around addiction—a factor which supported Nigel to reenter recovery following a previous relapse:

when I first went back there, I thought “bloody hell it’s going to be like walking back with your tail between your legs” you feel like such a failure. But nobody ever treats you that way when you go back, they’re just glad to see you again.

All participants spoke of the friendships they had formed through the organization, and the support that they had experienced. Shared pasts appeared to cultivate a sense of understanding, which helped participants to discuss struggles surrounding recovery: “It’s easier to talk to somebody that’s been in the same boat as you . . . ’cause they understand . . . they’ve gotta be in the same boat to understand how you’re dealing with it” (Michael). Feeling understood seemed to allow open communication, which fostered reciprocal emotional support and encouragement between the organization’s attendees. This social support stood to rebuild and broaden the social circles of all participants, with Michael explaining that “they’re the only people I associate with now, really—outside me family.” Collectively, this seemed to bolster feelings of belonging, to the extent that participants likened these social connections to familial bonds: “it’s like walking into a family home, that’s the best way I can put it. But a family home where nobody judges you” (Nigel); “We’re all one big family” (Sally). Collectively, feeling understood, unconditionally accepted, and supported led participants to reach out to the organization on a regular and routine basis for informal social interactions, or in times of crisis, offering an alternative coping mechanism to alcohol.

### Establishing a New Role in the Context of Recovery

All participants spoke of changes in their roles since entering recovery. All participants transitioned from being a recipient of support for their addiction, to being a provider of support for others with addictions. Typifying this, Sally acted as an interview panelist at a recovery center and emphasized that new staff “have got to learn what we need from them.” Similarly, Michael spoke of volunteering with an addiction center, and Nigel formed a music group for people battling addiction, as he believes that “the more [he] can help others, the better.” Collectively, participants placed themselves in a position of knowledge, expertise, and power on the road to recovery. Role models who had followed a similar path in recovery appeared to facilitate this role transition for Michael and Nigel. Nigel spoke of a musician who had also experienced addiction, explaining that his song lyrics resonated with his own addiction journey; a musician for whom he holds “admiration.”

Collectively, a change of role within the context of addiction appeared to foster participants’ self-confidence. This confidence led Sally to develop interests outside of her comfort zone: “I’ve done loads of speeches [about addiction recovery] . . . I would never do anything like that one time. [I had] no confidence and now me confidence. . . I go on holiday on me own now.” Similarly, Michael pursued qualifications to support others in recovery, attributing his confidence to return to university to new role models who had also experienced addiction prior to reentering education: “To see what other people have done, you just think, I could probably do this really.” In this vein, the participants’ change in role can be seen as altering their self-perceived occupational competence and contributing to new occupational identities within the world of addiction. In line with this, all participants spoke of having a new life and becoming a different person with changed priorities since cessation. Sally epitomized this change of identity that she tied to her new occupational roles and pursuits: “I like this Sally; I didn’t like the old Sally.”

## Discussion

This study has explored the occupational lives of individuals in sustained recovery from alcohol addiction. Findings reveal new meanings and purposes attached to occupational repertoires and roles throughout recovery, and that these are emerged through changes in engagement and are mediated by the environment. The value of establishing feelings of being understood, supported, and accepted also presented as integral to the participants’ continued recovery.

### The Nature, Meaning, and Purpose of Past Occupations

From an occupational perspective, the current study identified addiction as an isolating occupation by nature; it led to diminished social environments, relationship breakdowns and prevented meaningful, open connections with those remaining in participants’ lives. Within populations of working-age women, Luck and Beagan (2015) reported that addiction can foster social connections, which contribute to a sense of loss upon cessation. Furthermore, Narain et al. (2018) found that substance use did not inhibit participation in occupations, but it did affect the quality of that participation. The current findings suggest that social environments of all participants shrunk and were meaningless in the context of addiction, and they reported gaining social connections upon cessation. These differences may be attributable to the current participants’ attendance at the recovery institute which facilitated and cultivated strong bonds with others in recovery; it is possible that these connections led participants to reflect on their previous social networks as thin and meaningless relative to their newfound support.

Participants’ experiences highlighted that addiction was a meaningful past occupation; it held meaning and purpose as a strategy to cope with distressing thoughts and emotions. It also influenced the roles, routines, and behaviors of all three subjects, echoing the findings of Wasmuth et al. (2016). The participants’ occupational lives while addicted presented similar patterns to the findings of Andersson et al. (2012), whereby there was an absence of self-care and leisure occupations. Participants could be seen as experiencing occupational imbalance (Whiteford, 2000), which was only remedied when entering into recovery.

### The Meaning and Purpose of Current Leisure Occupations

In a mixed-methods study, Wasmuth and colleagues (2016) reported reengagement in leisure occupations to be linked with greater success in early recovery. The present findings expand upon this, highlighting leisure pursuits as supporting the later stages of recovery. Critically, leisure occupations served as new coping strategies in place of alcohol; findings therefore highlight the role, meaning, and purpose of leisure occupations in recovery. All participants articulated the enjoyment and therapeutic benefits they derived from their leisure pursuits. In the case of reengaging with past interests, participants appeared to have attached novel meanings, which brought about new purpose to their occupational engagement. Emblematic of this, Sally and Nigel reengaged with knitting and music, which became tools to support their mental health. Similarly, Michael reengaged with rugby as a co-occupation with his grandson. From an occupational perspective, this occupation can be understood as a conduit to developing a meaningful relationship that supports his recovery. This provides insight into the novel meaning and purpose attached to the reengagement in these once lost leisure occupations. Luck and Beagan (2015) likened recovery from addiction to an occupational transition, subject to acquisition of alternative ways to deal with stressors. The participants’ experiences illuminate how meaningful occupation can serve the purpose of coping with stress, provide tools to support recovery, and how this engagement influences participant perceptions of their mental well-being.

### The Influence of Social Environments on Recovery Maintenance

All participants highlighted accessing the organization as a key factor in their maintenance and indicated that it had been instrumental in increasing their social connections. A desire to form new friendships with others who share the goal of long-term recovery has been highlighted within treatment centers (Bell et al., 2015). The current study extends these findings into sustained maintenance, as all participants spoke positively of their new friendships with others in recovery. The participants suggested that it was important to be in an environment free of judgment, which allowed for members to be open, honest, and unashamed of their addictions. This evoked feelings of belonging, acceptance, and being understood. In 2008, Boisvert et al. developed a community for people in addiction recovery and delivered group interventions to foster group cohesion. The researchers found that if any member was to relapse, they were supported and encouraged to reengage with their recovery, rather than punished, judged, or banished. A similar occurrence appeared in the present study, whereby Nigel received unconditional acceptance and was welcomed to the organization following a previous relapse, which supported his reentry to recovery. Differing from Boisvert and colleagues’ findings, however, was the nature by which individuals formed these bonds and support networks; factors such as shared interests and shared pasts were shown to nurture these friendships in the present study, contrasting with group bonding exercises used by Boisvert and colleagues to cultivate these bonds. The present study provides further insight into the organic factors, such as common experiences, which can yield reciprocal, judgment-free social support in light of recovery, which is more representative of natural human behaviors.

### Occupational Adaptation in Sustained Recovery

All participants demonstrated that their position in the world of recovery had shifted substantially since their initial cessation. Indeed, they appeared to regard themselves as knowledgeable regarding the support that could help other people to enter and maintain recovery. This suggests that they no longer perceived themselves as someone who needed support, but as somebody who could offer support. Effectively, they had taken on a new role as a mentor to others in recovery. Changing one’s patterns of occupational engagement has been found to support early maintenance and treatment completion in addiction, and to lead to changes in occupational identity (Martin et al., 2011). The current findings demonstrate how these changes in occupational engagement and identity continue into sustained recovery. In line with this, the participants could be seen as experiencing occupational adaptation (Taylor & Kielhofner, 2017), as they perceived themselves as holding a positive self-identity and being competent within the environment of addiction and recovery. These findings should be used to inform future interventions that target the use of occupation for recovery (Leppard et al., 2018).

### Limitations

COVID-19 regulations resulted in the interviews being conducted via telephone, rather than in-person. Face-to-face interviews are often chosen to facilitate the collection of data that may be of a sensitive nature as they allow consideration of nonverbal communication (Lavrakas, 2008). Despite the present methodology relying solely on aural communication, some individuals find it easier to disclose personal information through methods of data collection that reduce the interviewer’s social presence (Schober, 2018). Telephone interviews may therefore have improved the richness of the acquired data. Study information and interviews were conducted in English, there is the possibility that this could have excluded potential participants. In keeping with the design of IPA research, the study’s sample was small and limited to one recovery center in Northern England. There can be a complex relationship between alcohol addiction and mental illness, identifying any past and current mental health problems of the participants could have brought further insights to the study. While this allowed for a purposive sample and homogeneity of participants, the transferability of these findings to wider populations could be limited. Potential participants were identified by a member of staff at the organization; although purposive sampling through a gatekeeper is a common method of obtaining a homogeneous IPA sample (Smith et al., 2009), there is a potential selection bias as the staff member may have selected individuals whose journeys to recovery may reflect positively on the organization (Denscombe, 2010).

## Conclusion

The findings from this research add to the body of research exploring addiction from an occupational perspective. Findings reveal the novel meaning, purpose, and role of reengaging in leisure occupations to facilitate recovery maintenance. The findings also illuminate progression into new roles which help to reconstruct positive self-perceived identities and capabilities inside and outside of the world of addiction. These findings suggest that nurturing patterns of occupational engagement and roles may support individuals to manage and maintain their recovery from addiction. Attention is drawn to the importance of occupational engagement and context in assigning new meaning. Applying an occupational perspective to long-term recovery maintenance may provide a new approach to supporting recovery which could complement existing pharmacological and psychological interventions (National Institute for Health and Care Excellence, 2011). Moreover, the current findings highlight the importance of forming friendships with others in recovery to foster feelings of belonging, acceptance, and reciprocal emotional support. In response, it is suggested that occupational therapists should facilitate the reestablishment of occupational engagement, and the development of social connections with others in recovery. Further research is warranted to explore the consistency of the current participants’ experiences with others in sustained recovery from addiction, including those with identified mental health difficulties and varying levels of occupational participation.
